# Conducting mental health studies in Emergency Departments in England: Lessons learnt from a randomised controlled trial testing a brief psychological intervention for adults who self-harm

**DOI:** 10.1371/journal.pmen.0000616

**Published:** 2026-05-14

**Authors:** Alexandra Elissavet Bakou, Giovanni Amati, Sally O’Keeffe, Tobit Emmens, Ashley Wilkie, Caroline Rowley, Rose McCabe

**Affiliations:** 1 School of Health and Medical Sciences, Department of Population Health and Policy, City St George’s, University of London, London, United Kingdom; 2 Population Health Sciences Institute, Newcastle University, Newcastle, United Kingdom; 3 Research and Development, Devon Partnership NHS Trust, Exeter, United Kingdom; 4 Mental Health Liaison Team, Coventry and Warwickshire Partnership NHS Trust, Coventry, United Kingdom; PLOS: Public Library of Science, UNITED KINGDOM OF GREAT BRITAIN AND NORTHERN IRELAND

## Abstract

Conducting mental health trials in busy EDs presents challenges, including record demand, bed shortages, 4-hour targets from arrival to discharge or admission, and staff shortages. This essay reflects on real-world challenges in a mental health trial involving people presenting to the ED with self-harm and/or suicidal thoughts and outlines lessons learned for future studies. No quantitative data will be reported. ASSURED is a randomised controlled clinical trial investigating the effectiveness of a brief psychological intervention for adults attending the ED with self-harm and/or suicidal thoughts. The original aims were to improve psychosocial assessments conducted by liaison psychiatry practitioners and to have the same practitioner deliver the intervention after discharge ensuring relational continuity. We worked with people with lived experience to address engagement challenges during mental health crises. 620 people across 14 EDs in England were recruited between 2022–2024. Although liaison psychiatry practitioners were trained to deliver the intervention, recruiting participants in crisis was difficult due to 4-hour targets and limited mental health research nurses. Delivering follow-up care post-discharge was challenging, as practitioners prioritised urgent assessments over delivering the follow-up intervention. However, it was feasible for researchers to recruit people within 1–2 weeks of ED attendance and for other mental health professionals to deliver the intervention. Flexibility in time and mode of delivery was paramount in participant engagement, which was mostly not achievable within liaison psychiatry roles. As many patients reattended the ED, experienced suicidal ideation or harmed themselves, supporting local clinical Principal Investigators to complete time-consuming paperwork in relation to these Serious/Adverse Events was helpful. ED liaison psychiatry teams face significant barriers to trial participation compared to other settings. Brief psychological interventions after ED attendance were more feasibly delivered by other practitioners such as assistant psychologists, and lived-experience advisors were central to ensuring sensitive engagement, approach and follow-up in research.

Clinical Trial Number

ISRCTN 13472559, Registered on 18 of November 2021.

## Background

Emergency Departments (EDs) play a critical role in mental health crisis management and access to services, often serving as the first point of care for individuals experiencing a crisis. In England, it is estimated that 4.2% of ED attendances are related to mental health crises [1]. Of these, an estimated 220,000 ED presentations are due to self-harm [2], resulting in approximately 100,000 hospital admissions each year [3]. Despite high levels of use, EDs are often described as poor environments for mental health care and the provision of a supportive and safe environment is critical to facilitating future treatment outcomes [4].

In the UK, death by suicide is a major public health concern; published data highlight increasing trends since 2005 [5–7]. Self-harm and suicidal thoughts are key risk factors for suicide [8]. The National Institute for Health and Care Excellence (NICE) defines self-harm as an intentional act of self-injury or self-poisoning irrespective of the apparent purpose of the act [9]. Risk of suicide following ED presentation with self-harm is high immediately following hospital discharge [10,11] and in many cases, self-harm occurs shortly before suicide with 15–43% of people attending the ED in the year before death [12,13]. Hence the ED is a key setting that provides an opportunity for assessment and early intervention [14].

In the U.K., when people present to the ED with self-harm, they are seen in the main ED for their medical needs and then referred to the (usually) co-located liaison psychiatry team for a biopsychosocial assessment. On average, ~ 60% of people attending the ED for self-harm receive a biopsychosocial assessment, which is typically carried out by a practitioner from the liaison psychiatry team who assesses the person’s problems and needs and arranges for appropriate aftercare [15,16]. There is a window of opportunity to support people when they present in crisis who may not avail of primary care or mental health services after the crisis has passed. However, recent research highlights a lack of interventions for people who present to the ED with self-harm and/or suicidality [17], and practical and ethical challenges in recruiting people who experience a mental health crisis present additional barriers to research in this field [18].

EDs are experiencing significant challenges such as record demand, bed shortages, 4-hour targets for discharge, and staff shortages [19]. It is estimated that in 2014 there were, on average, 61,318 ED attendances per day across the country and by 2019 this figure had risen to 70,230 attendances, an increase of 14.5% [20]. However, compared to other nations, the UK has a lower total number of hospital beds relative to its population and has experienced significant staff shortages [21]. As a result, the average time a person in mental health crisis spent in the ED in England was almost 11 hours [22,23]. In addition, several studies highlight an increased risk of psychological distress for staff working in the ED [24–26], often resulting in practitioners feeling powerless, particularly towards patients who present with self-harm and suicidality, leaving patients feeling judged and with a poor experience [27]. Developing, conducting and implementing a clinical trial for rapid psychological interventions in EDs for people presenting with a mental health crisis presents considerable challenges that may lead to early trial closure due to difficulties recruiting participants in this setting [28]. The aims of this paper is to describe the 1) organisational challenges and barriers in patient recruitment that we encountered and almost led to discontinuation of the trial and 2) practical solutions we developed and implemented to ensure continuation of the trial and 3) value of working closely with a Lived Experience Advisory Group (LEAP) during the trial. No quantitative data will be reported in this essay. Our reflections highlight the unique challenges of designing and conducting a mental health Randomized Controlled Trial (RCT) in busy ED settings in England and may provide valuable insights to researchers and clinicians designing, planning and conducting future trials testing psychological interventions in the ED setting.

## ASSURED Study at a Glance

ASSURED (‘Improving Outcomes in Patients Presenting with Self-Harm – Adapting and Evaluating a Brief Psychological Intervention in Emergency Departments’) aims to develop and test the clinical effectiveness and cost-effectiveness of a brief psychological intervention for adults who present to the ED with self-harm and/or suicidal thoughts [29]. London-City & East Research Ethics Committee provided ethical approval for the ASSURED study on the 12th of November 2021 (Reference: 21/LO/0683). The study was designed and developed collaboratively with a multidisciplinary team comprising lived experience experts, ED clinicians, ED liaison psychiatry practitioners, and academic experts in patient communication, suicide prevention, and trial methodologists. The study commenced in 2022, during the UK’s transition out of COVID-19 emergency measures. As a result, several adaptations were required to mitigate real-world challenges within a rapidly evolving context.

Patients eligible for the study were recruited and randomized to receive either Treatment as Usual (TAU) or the ASSURED intervention. Patients were eligible for inclusion if they were 18 years or older, presented to the ED, and were referred to the liaison psychiatry team for a biopsychosocial assessment. Eligible patients were those presenting with self-harm as defined by NICE [9] and/or suicidal ideation. All participants were required to provide written informed consent to take part in the study. Participants were excluded from the study if they were experiencing a psychotic episode, lacked the capacity to provide written informed consent, or required an interpreter due to the logistics involved in time-constrained EDs. Individuals who were Ministry of Justice patients subject to a restriction order, those living outside the NHS trust area, or cases where there were safety concerns were also excluded. The primary outcome is whether study participants re-attend ED and are referred to liaison psychiatry within 18 months from the date of randomisation. Secondary outcomes include suicidality, self-reported self-harm, psychological wellbeing, social outcomes, experiences of attending the ED, use of primary, community and social care services, and suicide. These are collected at 3,9 and 18 months post randomisation (for a full list of the outcomes, methods of collection and timelines, please refer to our protocol paper [29].

Based on previous findings on peoples’ experiences in the ED when presenting with self-harm and/or suicidality, the trial was designed for ED mental health practitioners to deliver the psychological intervention to make the biopsychosocial assessment more therapeutic in the ED and have relational continuity with the same practitioner after leaving the ED as continuity of care is emphasised by people experiencing a mental health crisis [30]. Based on evidence of effective components in brief psychological interventions after self-harm [31], the intervention includes an initial narrative interview and safety planning session, followed by up to four sessions of Solution-Focused Brief Therapy (SFBT). The person then receives three personalised letters from the practitioner ending at 9 months to remind them of their safety plan and support networks and an optional bank session. All participants provided written informed consent to participate in the study. The first patient in the study was recruited in the beginning of August 2022 and recruitment was completed on the 5^th^ of December 2024 across 14 ED departments in England. We describe and reflect on some of the challenges we encountered and some solutions to address these challenges that may be relevant for researchers planning to conduct mental health trials involving people in crisis in the ED.

## The capacity of liaison psychiatry teams to recruit participants and deliver a psychological intervention

Significant mental health staff shortages in the UK and worldwide have been extensively reported [32,33] and exacerbated since COVID-19, creating additional demands on staff in providing care [34] and engage in research activities [35]. For the ASSURED Study, o better understand the capacity to undertake research, we met 5–6 months *before* recruitment was scheduled to begin with the hospital Research and Development (R&D) teams and clinical leads, to establish relationships and assess the capacity of the liaison psychiatry team to participate in the study. Parameters identified to facilitate the study set up and participant recruitment initiation were: a) whether the study could pragmatically recruit a minimum of 8 liaison psychiatry practitioners locally to facilitate continuous supply of the intervention considering that mental health practitioners work on a rotation system (i.e., feasibility would be limited with smaller teams), b) whether a local Principal Investigator (PI) *and* a Sub-Investigator (Sub-I) with relevant research experience could be identified to lead the study locally, c) whether the services approached had access to appropriate treatment space (for example, clinical rooms that could be booked to ensure privacy and a quiet environment) and suitable audiovisual equipment to facilitate the optional delivery of the ASSURED intervention digitally, and d) assess the typical monthly flow of patients with relevant presentations Despite carefully considering these parameters it still proved difficult to recruit patients in the study according to the original study timelines; it took on average 8 months from reaching out to the local R&D team, to the first patient being locally recruited. Local leadership and alignment with the values and purposes of the study is pivotal for the clinical teams to engage and support research. However, liaison psychiatry clinicians involved in ED trials often struggle with the additional workload imposed by research activities. To effectively approach and identify PIs and Sub-Is to drive our study locally on hospital grounds, the team developed a short leaflet describing the study and the PI/Sub-I role: ensuring compliance with the research protocol (support with integrating the study procedures with the local day-to-day workflow of the service), safety reporting (liaising with the study researchers following a serious/adverse event, offering advice and guidance on managing safety concerns and resolving queries) and facilitating the intervention delivery. This provided clarity around the responsibilities, and the anticipated time and effort needed for the role. In addition, appointing a Sub-I (or multiple Sub-Is where possible) at the beginning of the study helped maintain continuity through staffing changes and ensured that the responsibilities were distributed. It was also important to support PIs in completing regular safety reporting paperwork related to Serious/Adverse Events. Serious/Adverse events reported more frequently included emotional distress, self-harm, suicidal thoughts that may indicate a risk and hospital admissions.

Recruiting liaison psychiatry staff to deliver the ASSURED intervention proved challenging due to shift patterns, staff shortages, and the lack of time for training and clinical work other than urgent biopsychosocial assessments. We developed a short leaflet with common practitioner questions and answers, encouraging liaison psychiatry staff to discuss with supervisors before deciding on whether they wished to participate.

Training liaison psychiatry staff in the ASSURED intervention was logistically challenging. Initially the study team planned to deliver two training events, each consisting of three consecutive days. However, this approach was not feasible for staff working on a rotating schedule, and it was difficult for liaison psychiatry team to release multiple staff simultaneously. Training was revised to be completed over two non-consecutive days instead of three, with sessions scheduled flexibly to accommodate liaison psychiatry staff availability. funding was secured to provide temporary clinical cover, which allowed practitioners to attend training without compromising service capacity. However, some teams did not have available bank staff. A digital training platform was developed to enable asynchronous learning and provide liaison psychiatry staff with continued access to the materials. In addition, twice-weekly, one-hour, drop-in supervision sessions were held to accommodate different shift patterns. Ensuring practitioners were available to deliver the intervention when a patient was recruited proved difficult. For liaison psychiatry staff, working in a therapeutic way over a longer period after ED attendance was a significant change from conducting a one-off biopsychosocial assessment in the ED. Liaison psychiatry teams do not usually offer follow-up appointments to adults presenting with self-harm and/or suicidal thoughts. This made the logistics challenging, including where and how to record clinical notes as usually the notes are closed when the person is discharged from the ED. One research site offered a follow-up clinic for people with complex needs or people who were seeking support more frequently, which supported this. Practitioners noted that a narrative interview might make the biopsychosocial assessment longer, impacting on time targets and how the follow-up SFBT sessions work within a liaison psychiatry team that is primarily tasked with assessment, signposting and onward referrals. Practitioners found it challenging to organize follow-up sessions while working on shift in the ED and prioritising urgent assessments and difficult to accommodate rearranging sessions for participants. In addition to the challenges with working on a rotational shift pattern, practitioners described challenges with accessing videoconferencing equipment/software, and a quiet space within a busy ED to deliver the intervention sessions. As a result, several practitioners could not deliver the intervention as planned [36]. Although we discussed dedicated spaces with EDs when planning the trial, the vast majority of sites were short of space and did not have access to other outpatient spaces. Future trials should integrate digital approaches so that people can be seen remotely to address this issue. To overcome these challenges, we worked closely with the Study Sponsor, the Study Funder and the local R&D teams to introduce dedicated research practitioner posts and devised a cross-cover intervention delivery model to deliver the intervention to patients remotely across different hospitals. Every practitioner providing cross-cover for another hospital site was assigned new patients as the study continued to recruit, rather than conducting sessions with patients already assigned. Therefore, even within this cross-cover model, we aimed to maintain practitioner continuity where possible. Dedicated practitioners were generally assistant psychologists (Band 5), funded by Excess Treatment Costs. The post was recruited to locally and the dedicated practitioner worked closely within liaison psychiatry teams. This ensured continuous supply of the intervention and uninterrupted participant recruitment. Finally, as the ASSURED study moved towards the later stages of the recruitment process (April 2024 – December 2024), a total of 13 ASSURED intervention practitioners (assistant psychologists and experienced clinical researchers) were recruited through various services of the participating research sites and directly supervised by more senior staff members of the participating liaison psychiatry teams. Though this mitigating factor altered the original study design and assumption that liaison psychiatry practitioners would deliver the ASSURED intervention within the ED and throughout the duration of the intervention delivery (8 weeks) [36], it represented a feasible adaptation that allowed us to protect the core components of the ASSURED intervention while ensuring that the full number of sessions could be delivered in a timely manner and within a short timeframe.

Lastly, patients in ED trials often face severe socioeconomic and logistical obstacles that reduce their likelihood of participation. For example, low-income patients may lack access to digital services and transportation, leading to higher rates of intervention non-attendance and study dropout as observed in other ED trials [37]. We mitigated this challenge by allowing for flexibility in the mode of contact and delivery of the intervention (the intervention could be delivered in person, over the phone or via videoconferencing) as well as provision to support patients with travel costs to attend the follow-up sessions. A summary of the real world challenges we came across and suggestions for future trials is presented in Table 1.

**Table 1:** Summary of Challenges and Suggestions for Future Trials.

Category	Challenges	Suggestions for future trials
**Prior to Participant Recruitment:** **Setting up research sites**	• Hospital capacity to undertake research	• Consider the necessary parameters (staff, clinical space, participant recruitment, resources needed) and discuss such parameters with local hospital R&Ds and Business Intelligence Teams 5–6 months in advance of recruitment.
**Recruiting and Retaining Practitioners**	Staff shortages in psychiatric liaison teams. Additional research workload disruptive for clinical work for participating practitioners and PIs. Practitioners may feel unsupported and research activities can be incremental to their work.	Discuss staff capacity to undertake research directly with the clinical teams. Identify and engage senior staff (nurses, psychologists, psychiatrists) for clinician buy-in. Consider how the research team can provide responsive and flexible supervision for the purposes of the study. Consider additional sources of funding to create protected time for research for participating practitioners. Consider a cross-cover intervention delivery model. Where possible recruit a Sub-I to support the lead PI with the research workload. Offer regular support to PIs for safety reporting.
**Training Practitioners**	• Difficulties in staff attending training due to shift rotations.	• Consider scheduling and flexible/multiple training dates as well as flexible modes of delivery (online delivery, asynchronous training).
**Screening, Consenting/Enrolling and retaining Participants**	Clinician-initiated referrals risked under-recruitment and the risk of selection bias. ED environment not conducive to informed consent for patients in mental health crisis Participant drop-out after revealing TAU allocation. Lengthy research follow-up periods	Consider extending screening responsibilities to local hospital researchers. Consider remote consent after people are discharge from the ED. Hold regular meetings with local hospital researchers to identify any bottlenecks in the recruitment process. Allow flexibility in contact mode and intervention delivery. Consider providing travel costs for participants where the intervention is taking place in-person. Work closely with people with lived experience to develop scripts for disclosing treatment allocation. Consider the use of self-report questionnaires where possible so that participants can have the option to complete them in their own time. Consider “light touch” communication with participants in-between follow-up researcher assessments.

## Improving recruitment and retention of participants who experience a mental health crisis

Studies relying on clinician-initiated referrals often risk under-recruitment due to staff clinical responsibilities [38]. The daily demands of liaison psychiatry practitioners made this challenging [36]. In addition, this approach renders the study susceptible to selection and recruitment bias. Initially, some avoided discussing the study with participants who attended the ED frequently as they felt that the intervention would be more suitable for people presenting to the ED for the first time. This also arose with patients considered too complex. The Lived Experience Advisory Panel (LEAP) and project team were committed to including as many people as possible to ensure equal opportunity to participate in research. The research team closely monitored the screening and enrolment process by reviewing screening logs and holding regular meetings with liaison practitioners to discuss cases.

When the study design was amended to recruit people after attending the ED (implemented in March 2023 – December 2024 see [36]), screening was undertaken by local hospital researchers. This ensured full screening of *all* referrals to liaison psychiatry against eligibility criteria rather than a pool of patients being screened when a participating practitioner was on shift. Hospital researchers approached participants remotely to discuss the study and obtain informed consent. Researchers held weekly meetings with hospital researchers to review screening notes and eligibility criteria. Participant withdrawal sometimes occurred during the allocation reveal (ASSURED vs. TAU), with participants randomised to TAU withdrawing from the study. Similar difficulties have been reported in other trials, particularly those recruiting participants during crises [39]. To address this, the research team worked closely with the LEAP to refine discussions around informed consent and develop scripts for disclosing allocation in a transparent yet supportive manner. This adaptation strengthened participant understanding of the trial design and helped mitigate attrition linked to disappointment following randomisation, particularly in the TAU group.

Maintaining follow-up engagement over an 18-month period proved to be one of the most demanding components of the study. Over this lengthy follow-up period, several participants moved to different areas in the UK or abroad, became uncontactable or had difficulties finding the time to carry out the researcher assessment. In addition, some participants described follow-up assessments as emotionally taxing, and shared worries around confidentiality and whether the disclosure of suicidal thoughts might negatively affect relationships or employment. In response, the trial implemented a series of adaptations driven by the LEAP. Study materials and questionnaires were revised (or abandoned) to ensure that language was accessible, non-judgemental, and less likely to elicit distress. Where possible, self-report measures replaced researcher-administered questionnaires, allowing participants to complete them at their own pace. Flexibility was embedded across all follow-up procedures: assessments could be completed by phone, video, or in person, and researchers offered evening or weekend appointments. Participants were sent questionnaires in advance if helpful to reduce uncertainty and help them prepare for the discussion. Furthermore, researchers offered to carry out the assessment over two days making the time commitment and emotional burden more manageable. To counteract the long intervals between follow-ups, the team prioritised relational continuity by assigning the same researcher to maintain contact with each participant whenever feasible. Light-touch engagement methods such as “staying-in-touch” texts and thank-you cards were introduced to maintain relationships with participants. Where consent was provided by the participants, researchers held regular meetings with hospital researchers to stay up to date with the most recent contact details of the participants.

## The importance of involving lived experience experts

With an increasing focus on person-centred care, working together with people with lived experience as well as people having cared for someone experiencing a mental health crisis is essential for the development and implementation of suicide prevention interventions [40]. ASSURED is rooted in the experience of people with lived experience who have been actively shaping, guiding and advising this program from the original study design, with a lived experience expert who is co-applicant on the funding application to the development of the intervention and dissemination of the research.

At the beginning of the ASSURED Program, a Lived Experience Advisory Panel (LEAP) was established and has since been actively involved in all stages of the study program to ensure that the production of knowledge and the research outcomes of the study are meaningful to participants and widely disseminated. Frequency of the LEAP meetings have been flexible depending on the phase of the study. The LEAP group has been actively involved in all stages of the study; from designing the study logo, conceptualizing and developing the study intervention and training materials, co-designing the research assessments, providing feedback on the communication practices between the study researchers and the study participants, as well as the dissemination of research findings and different stages of the implementation process. Crucially, LEAP input has been vital in detecting and problem-solving difficulties related to participant enrolment and retention in the study. The LEAP has been advising on different stages of the informed consent and the experience of consenting to research studies when experiencing a mental health crisis. In addition, the LEAP has been reviewing any research practices that may cause people to feel uncomfortable or distressed during a research assessment or interview. We have also had discussions around effective communication strategies for researchers, how often and when to approach participants for any follow-up data collection. Incorporating the unique lived experience perspective in our study, has been essential for the viability of our project.

## Conclusion and key takeaways

This paper provides insight into the barriers faced when running a multi-centre mental health trial of a psychological intervention for people presenting to EDs with self-harm and reflects on solutions. By providing insight into common difficulties which emerge by nature of the trial’s context as well as difficulties which are specifically tied to this clinical population, we hope to successfully drive patient engagement and methodological design of future ED trials working with patients experiencing suicidality and self-harm. ED trials require mutual understanding and high affinity between academic researchers, clinicians, people with lived experience and local R&D teams as well as a high degree of support to clinical teams, who already face the challenges of a pressurised clinical work environment. Lived experience involvement is crucial in informing research practices and navigating these challenges. Despite concerted efforts to preserve the original study design, the rapidly evolving post–COVID-19 context necessitated a number of difficult decisions regarding both the study design and the delivery of the intervention. This required careful judgement to strike an appropriate balance between safeguarding the core principles and integrity of the intervention while maintaining methodological rigour. The guidance and insight of our co-applicant clinicians and lived experience experts were instrumental in informing and supporting these decisions. Although ED trials can be challenging in their set up and recruitment for participants in a mental health crisis, the formation of an alliance between researchers, participants, lived experience experts and clinicians and an open dialogue between stakeholders was essential to successfully meet the recruitment targets of this study.
